# Aspirin for the next generation

**DOI:** 10.3332/ecancer.2013.300

**Published:** 2013-04-02

**Authors:** Nick Henderson, Tom Smith

**Affiliations:** 1 Aspirin Foundation, PO Box 223, Haslemere GU27 3ZJ, UK; 2 The Croft, Pinwherry, Girvan, Ayrshire, Scotland KA26 0RU, UK

**Keywords:** aspirin, cancer, dementia, diabetes, Hughes syndrome

## Abstract

First used as an analgesic and antipyretic, investigations into aspirin’s anti-inflammatory effects led to its establishment in 1974 as a drug that altered the activity of platelets to influence the course and incidence of myocardial infarction and cerebrovascular disease. It became the standard in treatment and prevention of vascular disorders.

The 25th International Scientific Meeting on aspirin held at the Royal College of Physicians in London on 24th October 2012 took aspirin into fresh fields, among them cancer, diabetes, dementia and gynaecology.

## Current trials involving aspirin

Dr Elmar Detering, of Bayer Pharma AG, Berlin, explained that there were currently 845 clinical trials of aspirin (www.clinicaltrials.gov), 145 of them using the low dose. The company has provided funding to 16 major studies of such subjects as the use of aspirin in primary prevention of diabetes, perioperative ischaemia in cardiac surgery, prevention of deep vein thrombosis in hip replacement, the patency of arteriovenous fistulae in patients with renal failure, colon cancer prevention, and secondary colon cancer.

Dr Detering’s colleague, Dr van Veenhuyzen spoke about the complexity of planning trials of aspirin in the prevention of colorectal cancer. The European Medicines Agency (EMA) confirmed, in September 2012, that there is a rationale for proceeding with trials and has endorsed the use of a geographical approach. Professor Rothwell is to define studies that will do this, at first in colorectal cancer, and perhaps, later in oesophageal cancer.

The Agency has accepted that aspirin’s long-term safety has been well established, but safety and all-cause mortality will still be scrutinised in the proposed trials. Dr van Veenhuyzen will work with Professor Rothwell and his collaborators to set timelines with the aim of placing a submission during 2013 and to start in 2014. The trials will be difficult: there will be many dropouts; many patients would need colonoscopies; and thousands of patients will require follow-up examinations for at least 15 years from the start of the trial.

## Aspirin in cancer

Dr Ruth Langley, of the UK Medical Research Council Clinical Trials Unit in London, spoke about the role of aspirin in treating cancer, a subject first mooted 40 years earlier from results in animal models. She showed data from a comprehensive review showing significant effects in colorectal and gastric cancer. The estimates for oesophageal cancer were similar to those for gastric cancer. There were also signs that aspirin may act on solid tumours such as breast, prostate and colorectal cancer.

In the last five years, three new pieces of evidence have awakened interest in aspirin and cancer. Fifty-one randomised trials were reviewed, that compared the use or nonuse of aspirin and other antiplatelet drugs, with almost 77,000 participants. There were fewer instances of cancer in subjects who had taken daily low-dose aspirin for five years or more, and a reduction of 15% in cancer deaths on aspirin. The reduction was not just in cancers of the gastrointestinal tract, but also in breast, prostate, and lung cancer. The effect was quickly evident and it was noted that aspirin also reduced the risk of metastases at diagnosis, as well as their subsequent development.

Patients with Lynch [1] syndrome (a hereditary predisposition to colorectal and other cancers) were randomised into taking either aspirin 600 mg or placebo. Those who had taken aspirin for more than two years were significantly less likely to develop cancer.

Epidemiological data on aspirin use after cancer diagnosis have shown that in colorectal cancer, regular aspirin use decreases both cancer and overall mortality. Data from breast cancer research been similar regarding the quite marked effects of aspirin on breast cancer deaths.

Dr Langley has proposed trials to see if regular aspirin use, after primary treatment in common solid tumours, will prevent tumour recurrence and prolong survival. It will involve four parallel trials in colorectal, breast, gastro-oesophageal, and prostate cancers. Her proposal has had wide support from the UK Oncology community physicians, patient groups, and from the National Cancer Research Institute clinical studies groups that prioritise cancer research in the UK. She is applying for grant funding and hopes to begin recruitment in 2013.

## Aspirin in the healthy elderly—ASPREE 

Professor John McNeil of Monash University, Melbourne, talked about the development of his work on aspirin in cardiovascular disease to its progression into geriatric studies and the development of ASPREE [2]. Six meta-analyses since 2010 have the advantage of six different endpoints—total mortality, non-fatal myocardial infarction, fatal myocardial infarction, serious vascular events, and ischaemic and haemorrhagic stroke.

Their results differed, and the benefit-risk of improved ischaemic stroke against increased cerebral haemorrhage was not clear cut. However, aspirin did appear to improve all-cause mortality; in the meta-analysis table, the effect of aspirin crosses the null line. This is largely driven by the non-vascular causes of death, mainly cancer. As the differences in cancer deaths emerge late in the studies, its anticancer effect may be greater than they suggest.

The group who would potentially benefit most from the use of aspirin are the elderly, as there may be a greater benefit for myocardial infarction than in younger ages. The potential trade-off includes more serious gastro-intestinal bleeds, more cerebral haemorrhages, and anaemia. ASPREE was originally a study in cardiovascular disease in the elderly (the patients were 70-plus years old), but as it evolved, it became a study of disability-free survival in which the patients were free from physical disability and cognitive impairment.

The study depends greatly on the specialist general practitioner. All the patients are aged 70 plus and are otherwise healthy; 16,000 Australian patients are expected to take part in this trial.

## Bleeding ulcers on aspirin: due to mucosal injury or to impaired haemostasis? 

Professor Chris Hawkey, of Nottingham University, based his study of aspirin as a cause of bleeding on it being of practical interest to clinicians. It satisfied an ambition to develop a way of doing studies in the community that would be inexpensive.

Aspirin use has been increasing and is the predominant cause of bleeding peptic ulcer in hospital admissions. In 2007, there were 2,833 peptic ulcer deaths, mostly due to bleeding, recorded by the Office of National Statistics. There were 12,864 hospital emergency admissions due to bleeding peptic ulcers, and meta-analyses gave a relative risk of 2.5 and a risk attributable to aspirin of 23%.

Professor Hawkey gave volunteers 300 mg aspirin a day for a month. A third of them had no mucosal injury, and another third had quite low levels of injury. This raised the question as to whether when low-dose aspirin produces problems, it is working, as he largely believes, through an anti-haemostatic effect, or through muscosal damage [3].

Professor Hawkey and Professor Neville Yeomans found that patients on aspirin who were H. Pylori positive had five times more risk of an endoscopic ulcer than those who were H. Pylori negative (23% and 4.9%, respectively). They hypothesised that aspirin causes bleeding from lesions that have other causes but are also attributed to H. Pylori. This differs from the evidence in NSAID studies, in which the associated increased risk of bleeding peptic ulcer was the same regardless of the presence or lack thereof H. Pylori.

They therefore proposed the Helicobacter Eradication Aspirin Trial (HEAT) in which the hypothesis is that NSAIDS directly cause ulcers and then bleeding, but that aspirin is not ulcerogenic in itself and instead increases bleeding when the cause of the ulcer is H. Pylori. A pilot study based on GP records addressed whether they were a valid and useful way of collecting endpoints. It showed a three-fold increase in upper gastro-intestinal bleeding on aspirin. There was a case, therefore, for doing the study. They contacted 2,500 patients and about a third were eventually tested. Twenty four per cent were positive for H. Pylori and underwent treatment, with a very high-eradication rate.

## HEAT 

On the assumption that a bleeding peptic ulcer on aspirin would occur at a rate of four per 1,000 (0.4%) per annum, and that there is a three-fold difference between patients who were H. Pylori positive and negative, the goal was to enroll 10,000 patients over two years. A total of 120,000 patients were written to, of whom 40,000 volunteered and out of those, 10,000 were H. pylori positive. The patients were randomised into treatment or placebo trials. This is all done by enthusiastic GPs and their practice teams.

In the pilot study, 47% of the patients replied, 33% took part in testing, 24% were H. Pylori positive, and in 93% of whom it was eradicated. This very high figure was surprising, but subsequently two reports have suggested that aspirin enhances H. Pylori eradication from about a typical rate of 70 to 90%.

The trial will allow comparison of methods of record collecting. Do GP records work well? How good are the Hospital Episodes Statistics (HES) and ONS data? And how reliable are patient self reports? Professor Hawkey has had support from the Comprehensive Local Research Network (CLRN) and its enthusiastic GP component. Sixteen CLRN nurses will each enroll 1,000 patients per annum, and thus over two years the trial should reach the target numbers. The trial covers half the country [4].

Based on 9.6 million elderly people in the UK, a quarter of them using aspirin and a quarter of them H. Pylori positive, if the HEAT has a positive outcome, this would lead to a decrease of 36,000 hospitalisations, and the overall number of lives saved would be 3,600 (240 per annum), at an estimated drug cost of under £5 per person.

## Aspirin and dementia 

Professor Antony Bayer of Cardiff University, and the Director of the Memory Team, University Hospital Llandough, spoke of his work with aspirin in dementia. The World Health Organization says that dementia is the biggest health and social services challenge of the 21st century. It is particularly challenging for the 80 and 90-year-olds, who have other co-morbidities. Delaying the onset of dementia by five years would halve its numbers. Aspirin seems an effective drug for its delay as vascular disease is part of many cases of dementia. For every case of dementia, the Caerphilly Prospective Study found two with milder cognitive impairment, so that the potential for prevention is huge.

Vascular disease, both individually and mixed with Alzheimer’s disease is very common, so if we had a treatment that reduces cerebrovascular disease it would help. However, vascular disease also plays a role in Alzheimer’s. In various dementia studies of postmortem examinations of older men, the brains showed that those with Alzheimer’s disease had a substantial amount of vascular pathology as well.

In the Caerphilly study, one of the problems is that during life the criteria for diagnosing dementia put a lot of emphasis on memory, so that they were designed to diagnose Alzheimer’s disease. In many prevalence studies, the mixed dementia group is placed among the Alzheimer’s numbers. Professor Bayer approached this slightly differently, so that his study’s mixed group contributed to slightly over half all cases of dementia.

There have only been two randomised controlled trials (RCTs) of aspirin in vascular cognitive impairment. One, which is nearly 25 years old [5] showed highly significant improvement in confusion and cognition on aspirin, but the numbers were very small, it was single blind, and randomisation was compromised. During this study, when subjects were unable to tolerate aspirin after randomisation, they were moved across into the control group. Nevertheless, the Alzheimer’s society in the UK promotes the use of aspirin on the basis of this study.

Richards *et al *in 1997 [6], also looked at the impact of aspirin but there were no baseline measures. However, they noted that verbal fluency and mental flexibility were significantly better among the patients on aspirin. These two characteristics reflect frontal lobe function and are often affected by small blood vessel disease. TPT involved 405 men aged 55 and over with CVD risk.

The Cochrane Collaboration concluded that no trials were eligible for review (it did not include either trial) and pointed out the difficulty of designing a trial today because there are ethical considerations in giving a placebo to patients with vascular dementia. Therefore, we have a common condition widely treated with a regimen for which there is little evidence.

However, there are observational data on the subject of aspirin in vascular dementia. Devine and Rands in 2003 [7] showed that people with dementia taking aspirin had an advantage in that they could stay at home for longer before being institutionalised and before death. As for primary care guidelines, aspirin is recommended in people with vascular dementia by the Scottish Intercollegiate Guidelines Network (SIGN) guidelines in Scotland. The National Institute for Health and Clinical Excellence (NICE) guidelines on dementia do not mention aspirin or antiplatelet treatment, but in practice in Canada and the UK, doctors do prescribe aspirin for vascular dementia.

Professor Bayer, as a geriatrician, reviewed many controlled trials of aspirin in dementia, but found problems with all of them. When over the age of 50-years-old, the subjects were at low risk of cognitive decline, so they were little different from the placebo group. If they did develop problems, they tended to drop out of the study or could not be bothered to do the tests, so they tended towards the null. The tests were inappropriate or insensitive; they could not be effectively done by telephone. Treatment was either too short or started too late, in that we realise that Alzheimer’s pathology develops 20–25 years before it gives the first signs of cognitive impairment. Therefore, we may need to institute effective prophylactic treatment 10 years before the symptoms arise.

He looked forward to the ASPREE study, which should overcome the objections to the earlier trials (such as the patients were too young and the outcomes were inappropriate) and provide the answers.

## Aspirin in pregnancy

Professor Graham Hughes, of St Thomas’s Hospital, and Editor of the journal *Lupus*, lectured on antiphospholipid syndrome, called by the media ‘sticky blood’ or Hughes syndrome. In 1975, when studying neuropathy in young Jamaican women, he found that they had circulating antibodies to phospholipids (aPL). Their main clinical problem was clotting; it produced the combination of repeated thrombosis and abortion, and cerebral disease. Sometimes, the first symptom, a DVT, occurs when a young woman goes on the pill for the first time.

Untreated women with Hughes syndrome have a very high risk of miscarriage, with 50% of losses in first trimester. Before effective treatment, only 19% of women with the syndrome carried their pregnancies to term. By 1996, among 60 pregnancies in women with Hughes syndrome, aspirin gave a live birth rate of 70%. It also reduced other complications such as pre-eclampsia, prematurity, fetal distress and intra-uterine growth retardation.

Professor Hughes picked out five patients to show the breadth of the syndrome. Among them were a man with spontaneous fracture in his foot; a woman of 51 with angina, previous deep vein thrombosis, three miscarriages, and clear coronary arteries on angiogram; a 21-year-old girl known to have Hughes syndrome, she was not on treatment and died from a coronary thrombosis; a 37-year-old woman with abdominal pain after meals, livedo, migraine from childhood, and deep vein thromboses; and a man with intermittent claudication, headaches, migraines with a family history of migraines and strokes.

The biggest and yet untapped symptom of Hughes syndrome is memory loss. Young women and young men say that they have ‘brain fog’, and worry that they may have dementia.

Antibody positive women who become pregnant are at high risk of pre-eclampsia, and of a syndrome of low-platelet counts and liver function abnormalities (HELLP syndrome). Aspirin dramatically helps the outcomes of such pregnancies (as does heparin).

A meta-analysis of five trials suggested that heparin plus aspirin led to a significantly higher live birth rate than either treatment alone. Most obstetricians give aspirin, but the additional use of heparin by self-injection daily is becoming more common. The women are very efficient in doing so, and many never have a headache for the whole nine months.

Professor Hughes would like every pregnant women to be tested for aPL syndrome, on the basis that 20% of recurrent miscarriages (over two miscarriages) are due to antiphospholipid antibodies. He also asks for long-term follow-up. There is evidence that they are at higher than expected risk of myocardial infarction and of stroke at an early age (before 45 years).

## Aspirin mode of action 

Professor Carlo Patrono of the Catholic University School of Medicine in Rome explained the action mechanisms of aspirin and the pharmacology of cyclo-oxygenase inhibition. Aspirin is not unique in inhibiting the cyclo-oxygenase pathways; other non-steroidal anti-inflammatory drugs can also do so but it is unique in the way it permanently inactivates the enzymes.

Today we know how aspirin works in preventing cardiovascular events. Acetylation of a single serine residue in the COX channel results in dose-dependent inhibition of platelet COX-1 activity, which can make a difference between life and death in acute myocardial infarction (as shown in ISIS-2 [8]). The low dose is all that is needed. The Anti-Thrombotic Trialists’ Collaboration meta-analysis of all the high-risk cardiovascular trials of aspirin showed that when it was used in the daily low-dose range, it produced a one-third reduction in the risk of major vascular events. When the dose was doubled, the reduction was one-fourth, and when it was ten times, higher the reduction was about one-fifth. Therefore, nothing is gained from an increased dose. If anything there could even be an inverse relationship between the daily dose and the clinical antithrombotic effect of aspirin [9]. Today, we would interpret this as reflecting a dose-dependent inhibition of vascular COX-2 activity and vascular prostacyclin production, which, as is known from the Coxib experience, can translate into a prothrombotic effect.

In vitro and animal studies have shown that products of the COX pathway, particularly prostaglandin E2, through interaction with four different receptors, can affect important target genes regulating growth, migration and invasion, angio-apoptosis and angiogenesis. All these responses can be down-regulated by aspirin. This has been confirmed by placebo-controlled randomised clinical trials in a clinical model of sporadic colorectal adenoma.

People who had had an adenoma surgically removed were randomised to a Coxib (celecoxib or rofecoxib) or placebo, or to low-dose aspirin or placebo. This was the trial that led to the withdrawal of Vioxx because of the emergence of cardiovascular toxicity. There were four other trials using doses between 81 and 325 mg once daily. Even the lowest aspirin dose gave a protective effect similar to that given by rofecoxib.

This may reflect an antiplatelet effect of aspirin impacting on platelet activation at sites of intestinal mucosal injury. Activation of platelets at such sites may release pro-androgenic and pro-inflammatory mediators, both lipid and protein in nature, that might cause endothelial and stromal cells in the intestinal mucosa to induce COX-2. This paradigm has been validated in vitro. It would lead to prostaglandin E2 production with its impact on angiogenesis, apoptosis, and cellular proliferation.

If this model is valid, it might explain the prevention of recurrence of a sporadic colorectal adenoma. Aspirin may act upstream by preventing platelet activation at the sites of intestinal mucosal injury, or acting downstream like traditional NSAIDs by inhibiting COX-2 activity.

Professor Patrono concluded that activated human platelets release a wide repertoire of lipid and protein mediators that may not only induce vascular occlusion, leading to myocardial infarction and ischaemic stroke, but also induce COX-2 in adjacent nucleated cells to produce colorectal carcinogenesis. Perhaps the same process of activated platelet release of inflammatory mediators includes amyloid beta-peptide and amyloid precursor peptide to induce neural degeneration. This is the possible basis for aspirin use in dementia.

## Putting side effects into perspective 

Professor Peter Elwood of the Cochrane Institute of Primary Care and Public Health, Cardiff, reviewed the current knowledge of the relationship between aspirin and bleeding. He wished to put it into perspective, because he is convinced that there is a very great difference between the perceptions of doctors and patients and the facts about bleeding by aspirin.

One of Carol Patrono’s studies showed that the bleeding risk by aspirin rises with age in people with no gastrointestinal symptoms, people with simple ulcer and people with complicated ulcer. The relative increase attributable to aspirin was 1.68 times the risk for patients on no aspirin in those with gastric pathology. Should such patients be on aspirin use or should they only be taking aspirin with a proton pump inhibitor (PPI)? Should they be tested for H. Pylori and if found to be infected, should the H. Pylori be eliminated first before being given aspirin?

How serious are the bleeds attributable to aspirin. In the first of the randomised controlled trials, the number of bleeds per year was 2.2 per 1,000 on low-dose aspirin and 1.6 per 1,000 on placebo, but the case fatalities of the bleeds on aspirin were actually lower than on placebo (four against five per 100,000 per year) suggesting that the bleeds provoked by aspirin were not the most serious.

In the Anti-Thrombosis Trialists (ATT) publication, the excess risk from aspirin was chiefly in non-fatal bleeds, and there were fewer fatal bleeds among participants allocated to aspirin than among the controls. In one of Peter Rothwell’s papers [10], the case fatalities from major extracranial bleeds were actually lower on aspirin than in controls. McQuaid and Laine [11] wrote that the risk amongst aspirin of any fatal bleeding was not significantly increased.

Deaths attributable to NSAIDs or aspirin use in hospital patients in Spain were 15 per 100,000, one-third of which were attributable to low-dose aspirin. Therefore, there are the same figures, five per 100,000 reported in the clinical trials. An US study of older veterans gives exactly the same figure of 5.5 deaths per 100,000. There is no room in these figures for any excess deaths due from bleeding attributable to aspirin.

In the 51 trial follow-up by Peter Rothwell, there was a high-excess risk from aspirin in the first three years that diminished quickly each year (relative risk of 1.95 after one year, and of 0.69 after five years), so that after five years there is no excess risk. Professor Elwood concluded that: gastro-intestinal bleeds are increased on aspirin; the bleeds attributable to aspirin are not the most serious; there is no evidence that aspirin induces fatal gastro-intestinal bleeds; and the excess risk reduces with time. He cannot yet define a risk–benefit balance between the effects of the bleeds and the benefit of aspirin on disease development. It has been well worked out in prevention of vascular disease, but these papers ignore the possible effect of aspirin on cancer. It would be of interest to see what the risk–benefit balance would be if the benefit of prevention of cancer were added to the benefit in cardiovascular disease.

The real disaster is cerebral bleeding. It is rare on low-dose aspirin with three to four events in 10,000 subjects. A major risk of spontaneous haemorrhagic stroke is high blood pressure. Therefore, it is appropriate to ask whether or not low-dose aspirin is likely to cause cerebral bleeding generally, or only in subjects with untreated hypertension. In the Hypertension Optimal Treatment (HOT) trial involving 10,000 subjects on aspirin, 10,000 on placebo, all hypertensive patients and given optimal treatment, there was no difference between the two groups in their incidence of haemorrhagic strokes, fatal or non-fatal. This has led to the suggestion that before patients or subjects go on long-term low-dose aspirin they should have their blood pressures measured.

Summarising, the evidence justifies that there is a serious mismatch between the perceptions about bleeding attributable to aspirin and reality.

## Aspirin in diabetes 

Dr Rajeev Raghaven, of the Wolverhampton Diabetes Centre, said that aspirin in diabetes has been a topsy–turvy topic in the last decade or so. People who would no longer prescribe aspirin in diabetes were enthusiasts a few years ago. Type 2 diabetes is predominantly a macrovascular disease and cardiovascular disease is the leading cause of morbidity and mortality in diabetes. Secondary prevention is a well-recognised area for the use of aspirin, which has achieved significant reduction in event rates. However, there is still the question of whether doctors are using treatment in the right ways. Is blanket treatment acceptable or should we be more nuanced in our approach to aspirin use?

We all have heard about aspirin use when there is an increased risk of cardiovascular disease, but this is even more pronounced in diabetes in which there is two to four times the risk of myocardial infarction, stroke and overall mortality than in the general population. Diabetes is amplifying the risk across the board, whether it is in cancer or cardiovascular disease. This is partly because the endothelium is a key organ affected by type 2 diabetes, and vascular inflammation underlies the disease process. In a population with increased obesity, there is an increased incidence of diabetes.

The rates of obesity, a true atherogenic and true inflammatory condition, are higher in the diabetes and prediabetes population. Trials in primary prevention have led to the major organisations in the field of diabetes advocating either limited or non use of routine aspirin.

In diabetes, there is now the term of aspirin ‘resistance’, possibly due to increased platelet turnover and decreased platelet responsiveness. In secondary prevention, aspirin use has been well supported, but the bleeding risk has been quoted time and again.

We are still not using aspirin in secondary prevention as much as we should; only 30% of patients who should be offered it in secondary prevention are in fact receiving it.

Dr Raghaven chose for discussion one review that looked specifically at studies of aspirin in primary prevention in diabetes. Most of the reductions in the risks crossed the unity line. The benefit appeared to be greater for myocardial infarction than for some of the other risks. In men, myocardial infarction was predominantly reduced; in women, the big reduction was in stroke risk. All cause mortality was not affected.

The AAA study in Edinburgh used 100 mg aspirin versus placebo, and there was no significant difference between the two arms in prevention of events. The gastro-intestinal event rate was slightly higher for aspirin than for placebo.

On this basis, the authorities in diabetes have shifted the age limit upwards from 40 to 50 for giving aspirin as primary prevention in diabetes. The US Physicians Health Study (USPHS) now advises a top level cut-off of 79 years for men and 70 years for women, and this may change with evidence from the ASPREE Trial. In Scotland, SIGN does not promote aspirin for primary prevention at all.

For secondary prevention in diabetes, Dr Raghavan advocates 100% aspirin use barring-specific contraindications. Hypertension may be a factor in whether or not aspirin is effective; in the Japanese Prevention of Atherosclerosis with angina in Diabetes (J-PAD) study, patients who attained significant lowering of their blood pressure were more responsive to aspirin than those who did not.

We need to be more specific about whom we target in diabetes rather than treating all diabetics either with aspirin or without because of the risk of gastrointestinal bleed. Dr Raghaven is concerned that more of his patients now are not taking aspirin because their primary care doctors are taking them off it. All patients over 40-years-old used to be given aspirin, but now it is largely confined to use in secondary prevention in older patients.

## Non-vascular effects of aspirin 

Professor Peter Rothwell of the University of Oxford has been interested, for the last 10 years, in the non-vascular effects of aspirin. Ten years ago Professor Rothwell was working on the follow-up of the UKTI Aspirin Trial, which studied aspirin in the secondary prevention of stroke, led by his mentor and boss Charles Warlow. They teamed up with Richard Peto and Richard Doll to look at long-term outcomes after the British Doctors Aspirin Trial. This was a primary prevention trial mainly focusing on cancer and on non-vascular outcomes. More recently, the collaboration has expanded to include most of the large aspirin trials including the alternate day trials with Nancy Cook and Mike Gaziano, as well as all of the large UK and European trials. Data were gathered from many smaller trials as well.

The aim of the non-vascular outcomes on aspirin collaboration (NoVA) is to put together all the individual patient data they can from previous and ongoing trials to look at the cancer, infection, and neurological outcomes that were not considered in the vascular trials.

They are hoping over the next few years to take forward the work that many people have done in the last 20 years. Subjects of this talk include cancer, other non-vascular outcomes, overall mortality and bleeding risk.

In an early meta-analysis of 19 case control studies, there was an impressive and consistent reduction in colorectal cancer among the patients taking aspirin. Meta-analysis of the non-randomised case control studies by cancer type (colorectal, biliary, oesophageal, etc.) which was then repeated with the long-term follow-up of the randomised trials, showed a similar pattern. The non-randomised studies do reflect the randomised trials, so, unusually, the observational studies are helpful because the randomised trials of the rarer cancers are never going to have enough power to give a reliable estimate of the effect of aspirin on their incidence or long-term mortality.

Due to this, various groups are continuing to collate the data from the non-randomised studies, and from the ongoing trials of aspirin.

## The 51 trials 

NoVA looked previously at the effect of daily aspirin versus control, on cancer deaths in 51 eligible trials of primary and secondary prevention in which patients had been randomised to aspirin for more than three months. While there is reduced cancer mortality on aspirin, after a five-year follow-up, the numbers of deaths are not large. There are 92 deaths due to cancer on aspirin, compared to 145 deaths without aspirin. These numbers should double with further follow-up of previous trials and addition of data from new trials in the next 5–10 years.

Most of this early difference in cancer mortality is probably due to an effect on metastasis. Many more data are needed to split up the deaths by type of cancer. The same applies to cancer incidence, in which there is a hint of a similar effect. So far, the numbers are too small to get a reliable estimate of effect even in the common cancers.

The NoVA group has studied the behaviour of cancers that presented in people who were taking aspirin before diagnosis and in those who were not. The effects of aspirin, in terms of reducing metastases at presentation in the primary prevention trials and on follow-ups are encouraging. The group still needs to add to these data because when they are split into the different types of cancer the numbers are not significant enough to say whether this is an effect that is generalizable to different cancers or is confined to, for example, gastro-intestinal and prostate cancers.

More data are also needed on the apparent aspirin effects on metastases and their impact on death due to cancer, comparing the aspirin and placebo groups in the trials.

A separate question is the effect of aspirin on long-term cancer incidence and mortality, which is a different effect from the effect on metastasis, and probably has a different mechanism. There is limited evidence of an effect on overall cancer incidence before five years, and no effect on colorectal cancer before 10 years, so there must be long-term post-trial follow-up to detect such effects. The NoVA team are trying to add data to the in-trial analyses with ongoing trials to increase the reliability of these effects beginning after five years. The long-term follow-up from the previous UK trials such as Scottish trial of Aspirin for Asymptomatic Atherosclerosis (AAA) and Prevention Of Progress of Arterial Disease And Diabetes (POPADAD) may give more information on long-term cancer incidence.

NoVA is also working with collaborators from the Women’s Health Study and the Physicians Health Study to compare the effects of the alternate day and daily aspirin, in long-term follow-up, to see whether the alternate day effects are similar to or smaller than those on daily aspirin.

There are hints that other cancers may be prevented by aspirin, too. The incidence of gastro-oesophageal cancer is also reduced on long-term follow-up, and there is possibly an effect on lung cancer, but the numbers so far are still too small. In the Women’s Health study with tens of thousands of patients, there were 16 oesophageal cancers, so combined analyses of data from many trials, perhaps including as many as 200,000 patients, are needed to gather the data needed for estimates of effect on specific cancers, particularly in low-risk groups such as young women who do not smoke.

There are observational data to suggest that aspirin might affect susceptibility to infection, diabetes, depression, suicide, various neurological diseases, valvular heart disease, and potentially all of these outcomes can be examined in the trial data.

## Discussion 

For Professor Rothwell, the biggest scandal is the lack of treatment of hypertension; a third of hypertension is diagnosed, a third of those diagnosed are treated, and a third of those treated are controlled. There is incredibly strong evidence that treatment of hypertension prevents strokes, heart attacks and dementia, but doctors do not do it.

Asked about general acceptance of the effect of aspirin on cancers, Professor Hawkey found that doctors who in a meeting accept that aspirin does have anticancer effects are reluctant to translate that into advice. He tells patients that on the balance of evidence aspirin does have a beneficial effect on some cancers, and advises on measures they can take to protect against the risk. It is not rocket science, he stressed.

Dr Tom Smith, GP and medical journalist, said that he had gathered from several talks that the two main risks for people taking aspirin, gastrointestinal and cerebral bleeds, might well be very significantly reduced by first eradicating H. Pylori infections and secondly by carefully controlling blood pressure. Was this not a message we could give to the medical profession and the public at large?

Professor Hawkey did not want that message to be made public yet. We do not yet have the evidence, particularly on H. Pylori. The current standard of care for people on aspirin who wish to avoid gastrointestinal bleeds is to use a PPI. If the message goes out too soon, it will undermine his trial, which will define whether or not patients really do need a PPI and to which groups it will apply. We cannot yet assume that H. Pylori eradication is a proven treatment.

Dr Langley was asked to summarise her work on aspirin and the treatment of cancer. Up to five years ago it was difficult to get the oncology community to become engaged with it, but three things have happened since then to stir up their interest.

The first was Peter Rothwell’s research on cancer outcomes in the vascular trials. The second was the outcome of the CAP-2 trial in patients with a hereditary predisposition to developing cancers; when they were randomised to placebo or aspirin, the latter group developed fewer cancers. The third was epidemiological evidence on aspirin use after cancer suggesting that it led to improved cancer mortality and overall mortality.

Putting these together, Professor Langley’s group is hoping to set up a large trial (ADASPIRIN) that will have cohorts of patients with colorectal, breast, gastro-oesophageal and prostate cancers, who will have had their primary treatment with curative intent. Those whose tumours recur will then be randomised to placebo or aspirin. Professor Langley hopes that aspirin will be an effective additional therapy for them.

Asked why we should not simply go ahead and give aspirin to cancer patients on the basis of what we already know about it, Professor Langley replied that it is difficult to introduce a therapy without a randomised trial to back it up. This trial aims to provide that evidence. It has a lot of support in the UK, so recruitment should go well, if the funding is made available. The trial is still in the planning stages.

Professor McNeil thanked the speakers for a tremendous meeting, and looked forward to the next one in Prato in 2014 where the discussion on aspirin will surely continue.
